# Association between chronic respiratory diseases and the attributable risk of long-term exposure to atmospheric particulate matters among middle-aged and elderly Chinese population

**DOI:** 10.1016/j.fmre.2025.04.013

**Published:** 2025-04-29

**Authors:** Wei-jie Guan, Shu-jun Guo, Li-lian Zeng, Jing Wei, De-jian Zhao, Rui-lin Meng, Yan-jun Xu, Yun-hong Lv, Chuan Li, Hong-ye Zhou, Jia-hui He, Li-feng Lin, Xue-yan Zheng

**Affiliations:** a Department of Allergy and Clinical Immunology, Department of Respiratory and Critical Care Medicine, State Key Laboratory of Respiratory Disease, National Clinical Research Center for Respiratory Disease, National Center for Respiratory Medicine, Guangzhou Institute of Respiratory Health, The First Affiliated Hospital, Guangzhou Medical University, Guangzhou 510120, China; b Guangzhou National Laboratory, Guangzhou 510163, China; c Guangdong Provincial Institute of Public Health, Guangzhou 511430, China; d Department of Atmospheric and Oceanic Science, Earth System Science Interdisciplinary Center, University of Maryland, College Park, MD 20740, USA; e Guangdong Provincial Center for Disease Control and Prevention, Guangzhou, 511430, China

**Keywords:** Chronic respiratory diseases, Coarse particulate matter, Fine particulate matter, Submicron particulate matter, Disease burden, Population attributable risk

## Abstract

Chronic respiratory diseases (CRDs) mainly consist of chronic obstructive pulmonary disease (COPD), asthma and chronic bronchitis. The attributable risks of CRDs associated with long-term exposure to ambient particulate matters (PMs, aerodynamic diameter ≤1, 2.5 and 10 µm: PM_1_, PM_2.5_ and PM_10_) are unclear, particularly in developing countries. We determined the association between long-term exposure to PMs and CRDs in the middle-aged and elderly Chinese population and their corresponding population-attributable fractions (PAF). We conducted a *COPD Surveillance* study in Guangdong, China. We derived the averaged 722-day exposure estimates of PM_1_, PM_2.5_ and PM_10_ at 1 km × 1 km resolution at individual residential addresses between 2013 and 2019. The associations between PMs and CRDs were estimated by logistic regression models. We calculated the PAF of CRDs attributable to long-term PM exposures. We included 7371 participants aged 40 years or greater, among whom 1430 (19.40%), 736 (9.99%), 731 (9.92%) and 313 (4.25%) had CRDs, COPD, chronic bronchitis and asthma, respectively. Each 10 µg/m^3^ increment of PMs was positively associated with the proportion of patients with CRDs, COPD, chronic bronchitis and asthma. The associations were more prominent in participants residing in rural areas and exposed to biomass fuel. The PAF of CRDs associated with PM_1_, PM_2.5_ and PM_10_ was 19.09% (95%CI: 12.41%∼25.02%), 18.98% (95%CI: 12.90%∼24.90%) and 21.40% (95%CI: 13.84%∼27.67%), respectively. The PAF of COPD associated with PMs was highest, followed by asthma and chronic bronchitis. In summary, ambient PM exposure is linked to a heightened risk of CRDs and accounts substantially for CRDs among the middle-aged and elderly population.

## Highlights

• This study determines the attributable risk of long-term PMs exposure to CRD.
• Novel PMs estimate models have been developed to accurately assess individuals exposure.
• Prolonged PM exposure is linked to a heightened risk of CRDs in middle-aged and elderly individuals.
• Residents in rural areas and exposed to biomass fuels exhibit more prominent associations.
• The PAF of COPD associated with PMs is highest, followed by asthma and chronic bronchitis.

## Graphical abstract

**Figure:**
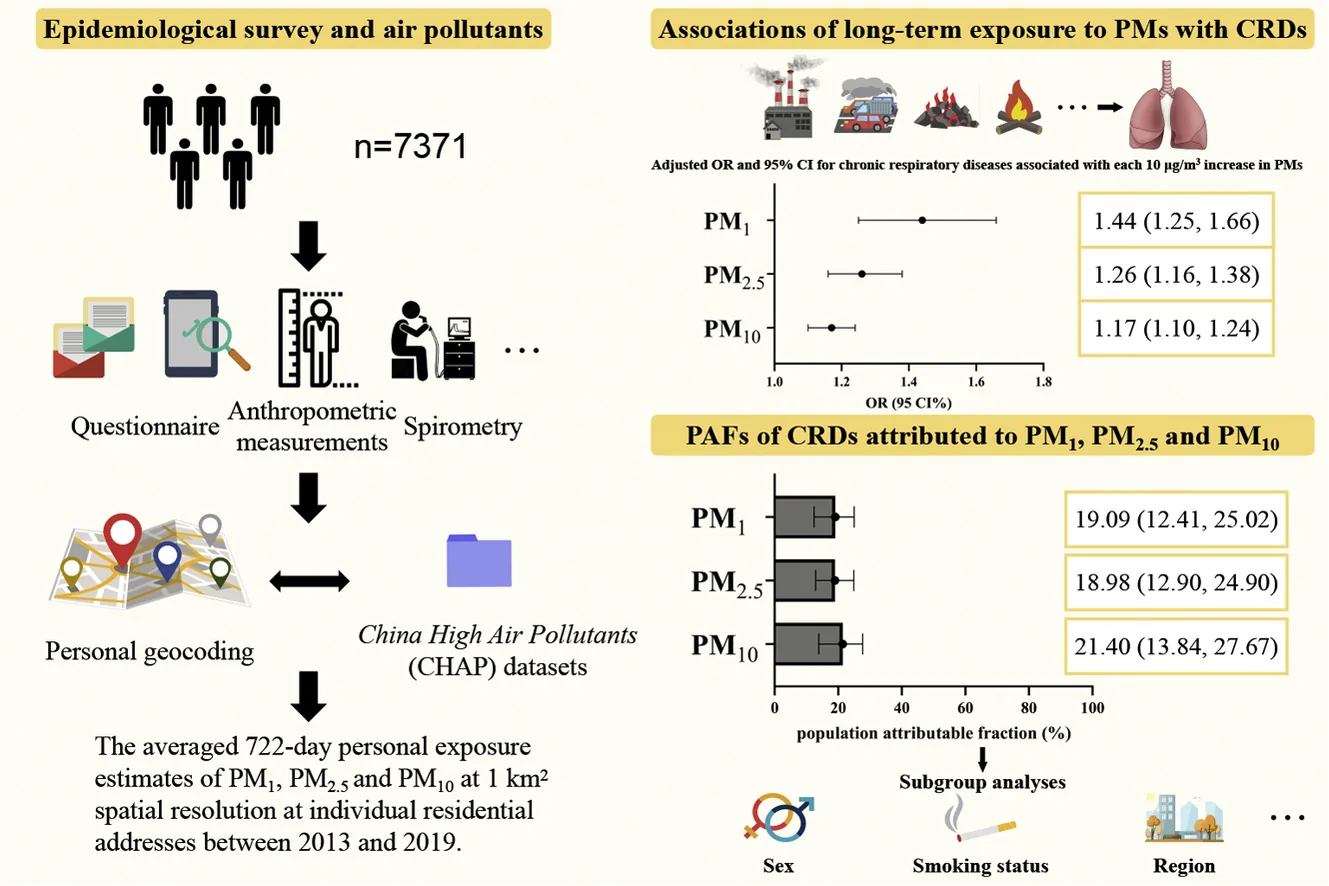

## 1 Introduction

Chronic respiratory diseases (CRDs) have been the third leading cause of death worldwide, representing a growing public health issue [1]. Developing countries had the highest mortality and disability-adjusted life years of CRDs [1,2]. The major burden of CRD in low- and middle-income countries has been attributed to smoking globally, followed by air pollution [1]. Despite the widespread concerns, the associations between CRDs and their attributable risk of air pollution have not been thoroughly investigated [3,4].

Accumulating evidence has shown that air pollution is associated with poorer symptom control of CRDs [1], such as chronic obstructive pulmonary disease (COPD), chronic bronchitis (CB) and asthma [[5], [6], [7], [8], [9]]. However, understanding the attributable risk of CRDs associated with air pollution would provide policymakers with valuable insights into the potential for disease prevention. This information is crucial for implementing effective public health strategies and preventive measures [10]. This could be achieved via the calculation of the population-attributable fraction (PAF), which represents the proportion of the disease in the population that can be attributed to a certain risk factor. PAF quantitatively describes the health impact of a certain risk factor within the population.

Previous studies profiling the burden of CRDs attributable to air pollution have focused on specific disease conditions (e.g. COPD and asthma) [[11], [12], [13], [14]]. However, there remains a paucity of evidence documenting the burden of CRDs as a whole, which is attributable to exposure to particulate matters (PMs, aerodynamic diameter ≤ 1, 2.5 and 10 µm: PM_1_, PM_2.5_ and PM_10_) [15]. Little is known about the long-term effects and attributable risks of PMs on CB, the CRD that might predate COPD. Most existing evidence is derived from other developed countries or regions, and studies examining the impact of PM concentrations in regions of China where levels are particularly high remain limited. Although the average PM_2.5_ concentration across China in 2021 has been significantly reduced to 30 µg/m^3^, it remains 6 times higher than that recommended by World Health Organization air quality guidelines 2021 (5 µg/m^3^). Furthermore, Guangdong is one of the most economically developed provinces in China, contributing nearly one-fourth of the national GDP, yet it still faces significant regional development imbalances, which is a microcosm of China as a whole. Hence, it would be imperative to elucidate the attributable risk of long-term ambient PM exposure for CRDs and the proportion of patients explained by each type of PMs in Guangdong, China.

Here, we estimated the associations between ambient PMs and the risk of CRDs among the middle-aged and elderly population in South China based on a cross-sectional study. We further estimated the burden of CRDs attributable to PMs by using a health risk assessment framework.

## 2 Methods

### 2.1 Study design

The details of sampling have been published previously and are available in the *Online Supplement* file. Briefly, a probability-based multi-stage sampling strategy was employed at six surveillance sites in Guangdong province, China. The surveillance sites included: Doumen District (Zhuhai), Panyu District (Guangzhou), Yingde (Qingyuan), Luoding (Yunfu), Taishan (Jiangmen) and Wengyuan County (Shaoguan). We included adults aged greater than 40 years in the 2014–2015 and 2019–2020 Chronic Obstructive Pulmonary Disease Surveillance (COPD surveillance) in Guangdong [16,17]. Residents who had lived in their current residence for >6 months within the survey year were included. Residents with malignancy, paraplegia, cognitive defects, linguistic or mental disorders, and pregnant or breastfeeding women were excluded. During the COPD surveillance, data were collected in a healthcare facility by trained staff from the local health stations or community clinics. Participants should complete: questionnaire, anthropometric measurements (height, weight, waist circumference, blood pressure, heart rate) and spirometry [by trained staff by applying a nose clip and using commercial spirometer (MasterScreen Pneumo, Jaeger, Germany), based on international guidelines] [18,19]. We performed a bronchial dilation test with 400 µg salbutamol (GlaxoSmithKline, Middlesex, UK) via a spacer. Quality control was conducted with the five-level grading scheme based on the National Lung Health Education Program (grades A-F). We obtained at least three technically acceptable and repeatable maneuvers and included the maneuvers with grades A, B and C (the difference between the best two readings of FEV_1_ and FVC within 200 mL) [20].

The Ethics Review Committee of the National Center for Chronic and Non-Communicable Disease Control and Prevention, China Center for Disease Control and Prevention approved the study protocol (No. 201410 and No. 201901). Written informed consent was obtained from all participants.

### 2.2 Clinical assessment

Clinical endpoints included CRDs, COPD, asthma and CB. We used the GOLD criteria to identify individuals with COPD, whose post-bronchodilator FEV_1_/FVC <70% [21]. Although COPD should be suspected in individuals with symptoms and exposure to noxious stimuli, we used the simpler criterion for defining COPD [22,23]. Asthma denoted a self-reported history of asthma diagnosis by a physician or wheezing in the preceding 12 months [24]. CB was defined as coughing up phlegm for at least three months in two consecutive years [25]. Respondents also reported the frequency of respiratory symptoms. We defined CRDs as participants diagnosed as having COPD, asthma or CB [26,27].

### 2.3 Air pollutants

The China High Air Pollutants (CHAP) datasets were used to obtain the daily concentrations of ambient PM_1_, PM_2.5_ and PM_10_ at 1 km × 1 km (km^2^) spatial resolution from 2013 to 2019. Using the Baidu Maps API (http://lbsyun.baidu.com/), the home locations of each participant were geocoded to match the geocodes of each pollutant from the CHAP datasets. The initial participant was enrolled on Dec 23, 2014, while PM concentrations were accessible from the earliest date (Jan 1, 2013) by matching the related geocode in the CHAP datasets. Because long-term PM exposure was usually defined among participants who have been exposed to air pollutants for more than one year [28,29], we chose the exposure window of 722 days (the time frame with all available data) and studied the associations of the 722-day mean exposure levels of PMs with CRDs. The PM_1_, PM_2.5_ and PM_10_ estimates have been discussed previously [[30], [31], [32]]. Briefly, the daily time-series dataset of PM_1_, PM_2.5_ and PM_10_ concentrations at 1 km^2^ grids in China from 2013 to 2019 in China was created using a space-time extremely randomized tree model. During the production of the ChinaHighPM_1_/PM_2.5_/PM_10_ datasets, the data as the model input mainly included the satellite remote sensing (MAIAC AOD), multi-resolution emission inventory for China pollution emissions, meteorological characteristics, land use and urban form (land use, road, population), and the spatiotemporal terms. A time-series and full-coverage dataset of PM_1_, PM_2.5_ and PM_10_ at 1 km^2^ cell grids was produced. According to 10-fold cross-validation [[30], [31], [32]], greater consistency of the spatiotemporal autocorrelation could be observed between the estimated PMs [coefficient of determination (*R*^*2*^): 0.83, root-mean-square error (RMSE): 6.2 µg/m^3^], PM_2.5_ (*R*^*2*^: 0.92, RMSE: 10.8 µg/m^3^), PM_10_ (*R*^*2*^: 0.90, RMSE: 21.1 µg/m^3^) and ground-level measurements at daily scales. To estimate the PMs concentration, geocoding was performed at the residential location of study participants. This enabled the calculation of the average exposure of each participant in the 722 days prior to the survey.

### 2.4 Confounding factors

A standardized questionnaire was employed to capture the information to examine the potential confounding factors. We captured the following covariates from the questionnaire: sex (males, females), age, residential region (urban, rural), educational level (primary school or lower, middle school, university education or higher), marital status (unmarried, married, widowed or divorced), household ventilation (yes, no), biomass fuel exposure (yes, no), smoking status (ever smoker, never smoker), passive smoking (yes, no), occupational exposure (yes, no) and body-mass index (BMI) category (underweight/normal weight, overweight/obese).

### 2.5 Statistical analysis

#### 2.5.1 Associations between PMs and CRDs

The initial analysis of the concentration-response relationship of PMs with multiple CRDs suggested almost linear positive association, according to the natural spline smoothing function [33] (Fig. S1). Our initial analysis revealed a near-linear effect of PMs and CRDs, we therefore selected the lowest concentration as the threshold. Next, we investigated the associations between PM concentration and CRDs from the fully included population using multivariate logistic regression models. We included the covariates that consisted of the age, educational level, marital status, BMI category, region, passive smoking, occupational exposure, household ventilation, biomass fuel exposure, smoking status and sex to control for confounding effects, which were selected in the model and had statistically significant or clinical associations with CRD, COPD, asthma or CB. In addition, we tested the collinearity among air pollutants and covariates using the Variance of Inflation (VIF) Score. The VIF of 5 or greater indicated collinearity among the variables. Variables with significant collinearity were excluded due to multicollinearity. Specifically, PM_1_, PM_2.5_, and PM_10_ exhibited significant collinearity (VIF > 5) with each other and with other pollutants (except O_3_). Therefore, each model included only one pollutant to avoid multicollinearity.

The odds ratios (ORs) and 95*%* confidence intervals (95%CIs) were estimated for 10 µg/m^3^ increment in PM concentrations in a single-pollutant model.

#### 2.5.2 Estimating attributable CRD burden

Based on the above-mentioned estimates, we calculated the PAF of multiple CRDs due to ambient PMs exposure based on the following formula:

$$PAF=\left[\sum \left(P_{i}\times RR_{i}\right)-1\right]/\left[\sum \left(P_{i}\times RR_{i}\right)\right]$$

where Pi indicated the proportion of population in the exposure category “i” (using each PM_1_, PM_2.5_ and PM_10_ concentration; 1 µg/m^3^ as the category) and RR_i_ represented the OR of exposure category “i ”. Because RR approximated the OR within the cross-sectional study, we adopted the OR for PAF calculation.

#### 2.5.3 Stratified and sensitivity analysis

To identify potential confounding factors, we conducted stratified analyses with sex, age, cigarette smoking, biomass fuel exposure, occupational exposure, ventilation, BMI category and residential region. We also introduced a cross-product term to examine the statistical interactions between long-term PM exposure and confounders.

The statistical significance for the between-group differences was examined by calculating the 95%CI as

$$\left(\beta 1-\beta 2\right)\pm 1.96\sqrt{{\text{se}}_{1}^{2}{+\text{se}}_{2}^{2}}$$

where β_1_ and β_2_ were the coefficient estimates for each stratum, and se_1_ and se_2_ were the standard errors.

We conducted a sensitivity analysis that excluded cardiovascular diseases (cor pulmonale, coronary heart disease, arrhythmia, hypertension, and stroke) and other respiratory diseases (rhinitis, allergic rhinitis, pulmonary arterial hypertension, tuberculosis, and lung cancer), in light of their significant associations with PMs [15].

We conducted analyses using R software, and *P* < 0.05 was considered statistically significant.

## 3 Results

### 3.1 Study population and CRDs

Of 7510 participants screened, 139 were excluded due to missing data. Specifically, 3 participants did not respond to symptom items and 136 did not indicate their height. Therefore, 7371 participants were finally included. The total and included participants had comparable characteristics (Table S1). The proportion of patients with CB (9.92% vs. 10.03%) and asthma (4.25% vs. 4.33%) was well balanced between the included participants and the total participants. The concentrations of PM_1_ (20.85µg/m^3^ vs. 20.82 µg/m^3^), PM_2.5_ (34.92 µg/m^3^ vs. 34.88 µg/m^3^) and PM_10_ (54.35 µg/m^3^ vs. 54.30 µg/m^3^) were similar.

Among the included participants, 1430 (19.40%) had CRDs. Participants with CRDs were older, more likely to be males and underweight, had lower education levels, have lived in rural areas, and were more frequently exposed to biomass fuel, cigarette smoke and occupational pollutants compared with the participants without CRDs. The 722-day mean PM_1_, PM_2.5_ and PM_10_ concentrations were 21.45µg/m^3^, 35.87µg/m^3^ and 55.60µg/m^3^ among participants with CRDs, which were significantly higher than those among participants without CRDs. Details of the included participants with CRDs are demonstrated in Table 1. Summary statistics and Spearman correlations of the 722-day mean air pollutants are shown in Table S2.

**Table 1:** **Demographic and characteristics of the participating of chronic respiratory diseases**.

Characteristics	All (No. 7371)	Chronic respiratory diseases participants (No. 1430)	COPD participants (No. 736)	Chronic bronchitis participants (No. 731)	Asthma participants (No. 313)
**Social demographic indices (SDI)**					
**Sex, *n* (%)**					
Male	3764 (51.06)	1033 (72.24)	610 (82.88)	522 (71.41)	172 (54.95)
Female	3607 (48.94)	397 (27.76)	126 (17.12)	209 (28.59)	141 (45.05)
**Age (year), mean (SD)**	57.26 (9.26)	60.62 (9.37)	62.99 (8.74)	59.79 (9.62)	59.75 (9.74)
**Educational level, *n* (%)**					
None + primary school education	4231 (57.40)	843 (58.95)	428 (58.15)	422 (57.73)	215 (68.69)
Middle school education	2989 (40.55)	570 (39.86)	302 (41.03)	300 (41.04)	93 (29.71)
University education or higher	151 (2.05)	17 (1.19)	6 (0.82)	9 (1.23)	5 (1.60)
**Marriage status, *n* (%)**					
Unmarried	94 (1.28)	23 (1.61)	12 (1.63)	10 (1.37)	6 (1.92)
Married	6720 (91.17)	1304 (91.19)	669 (90.90)	673 (92.07)	281 (89.78)
Widowed or divorced	557 (7.56)	103 (7.20)	55 (7.47)	48 (6.57)	26 (8.31)
**Region of residence, *n* (%)**					
Urban	2745 (37.24)	497 (34.76)	237 (32.20)	267 (36.53)	125 (39.94)
Rural	4626 (62.76)	933 (65.24)	499 (67.80)	464 (63.47)	188 (60.06)
**Household air pollution exposure, *n* (%)**					
**Household ventilation**					
No	1536 (20.84)	314 (21.96)	191 (25.95)	155 (21.20)	69 (22.04)
Yes	5835 (79.16)	1116 (78.04)	545 (74.05)	576 (78.80)	244 (77.96)
**Passive smoking**					
No	3171 (43.02)	603 (42.17)	311 (42.26)	307 (42.00)	131 (41.85)
Yes	4200 (56.98)	827 (57.83)	425 (57.74)	424 (58.00)	182 (58.15)
**Biomass exposure**					
No	4633 (62.85)	846 (59.16)	441 (59.92)	434 (59.37)	176 (56.23)
Yes	2738 (37.15)	584 (40.84)	295 (40.08)	297 (40.63)	137 (43.77)
**Smoking status**^a^					
Never smoker	4188 (56.82)	470 (32.87)	159 (21.60)	257 (35.16)	148 (47.28)
Ever smoker	3183 (43.18)	960 (67.13)	577 (78.40)	474 (64.84)	165 (52.72)
**Occupational exposure**					
No	3695 (50.13)	645 (45.10)	368 (50.00)	303 (41.45)	135 (43.13)
Yes	3676 (49.87)	785 (54.90)	368 (50.00)	428 (58.55)	178 (56.87)
**Anthropometry**					
**BMI category**					
Normal weight or underweight	3979 (53.98)	855 (59.79)	504 (68.48)	420 (57.46)	154 (49.20)
Overweight or obese	3392 (46.02)	575 (40.21)	232 (31.52)	311 (42.54)	159 (50.80)
**Ambient air pollutants exposure**					
PM_1_	20.85 (4.34)	21.45 (4.36)	21.78 (4.34)	21.17 (4.36)	21.61 (4.26)
PM_2.5_	34.92 (6.97)	35.87 (7.02)	36.30 (6.92)	35.53 (7.13)	36.23 (6.79)
PM_10_	54.35 (9.89)	55.60 (9.86)	56.13 (9.62)	55.15 (10.11)	56.35 (9.68)

a Ever smoker included current smoker and former smoker.

### 3.2 Associations of long-term exposure to PMs with CRDs

We observed almost linear positive associations of PM_1_, PM_2.5_ and PM_10_ with multiple CRDs (Fig. S1). Each 10 µg/m^3^ increment of PMs was positively associated with the risk of CRDs [PM_1_ (OR: 1.44, 95%CI: 1.25∼1.66), PM_2.5_ (OR: 1.26, 95%CI: 1.16∼1.38) and PM_10_ (OR: 1.17, 95%CI: 1.10∼1.24)] (Table 2), COPD [PM_1_ (OR: 1.85, 95%CI: 1.53∼2.24), PM_2.5_ (OR: 1.45, 95%CI: 1.29∼1.63) and PM_10_ (OR: 1.28, 95%CI: 1.17∼1.39)] (Table S3), CB [PM_2.5_ (OR: 1.12, 95%CI: 1.00∼1.25)] (Table S4) and asthma [PM_1_ (OR: 1.33, 95%CI: 1.03∼1.72), PM_2.5_ (OR: 1.22, 95%CI: 1.04∼1.43) and PM_10_ (OR: 1.17, 95%CI: 1.04∼1.31)] (Table S5) after adjusting for the covariates which had statistically significant or clinical associations with CRD, COPD, asthma and CB.

**Table 2:** **Adjusted OR and 95% CI for chronic respiratory diseases associated with each 10 µg/m^3^ increase in PMs by strata**.

Strata	No. of participants	Chronic respiratory diseases					
		PM_1_		PM_2.5_		PM_10_	
		OR (95% CI)	*P* for difference test^a^	OR (95% CI)	*P* for difference test^a^	OR (95% CI)	*P* for difference test^a^
**Total**	7371	1.44 (1.25, 1.66)	–	1.26 (1.16, 1.38)	–	1.17 (1.10, 1.24)	–
**Sex**							
Male	3764	1.52 (1.27, 1.81)	–	1.29 (1.16, 1.44)		1.18 (1.09, 1.28)	–
Female	3607	1.38 (1.08, 1.75)	0.50	1.25 (1.08, 1.45)	0.69	1.17 (1.05, 1.30)	0.78
**Age**							
40–50	1776	1.28 (0.91, 1.79)	–	1.21 (0.98, 1.48)	–	1.18 (1.01, 1.37)	–
50–60	2747	1.40 (1.09, 1.78)	0.70	1.24 (1.06, 1.44)	0.88	1.14 (1.03, 1.27)	0.76
≥60	2844	1.65 (1.34, 2.03)	0.22	1.36 (1.20, 1.55)	0.36	1.22 (1.11, 1.34)	0.67
**Smoking status**							
Never smoker	4188	1.25 (1.00, 1.56)	–	1.17 (1.02, 1.35)	–	1.11 (1.01, 1.23)	–
Ever smoker	3183	1.62 (1.35, 1.96)	0.06	1.35 (1.20, 1.51)	0.13	1.22 (1.12, 1.32)	0.16
**Biomass fuel exposure**							
No	4633	1.35 (1.13, 1.60)	–	1.21 (1.09, 1.35)	–	1.14 (1.06, 1.23)	–
Yes	2738	1.67 (1.28, 2.18)	0.21	1.39 (1.18, 1.63)	0.16	1.23 (1.09, 1.38)	0.33
**Occupational exposure**							
No	3695	1.53 (1.23, 1.89)	–	1.31 (1.15, 1.50)	–	1.17 (1.07, 1.29)	–
Yes	3676	1.36 (1.13, 1.65)	0.46	1.22 (1.09, 1.37)	0.45	1.16 (1.07, 1.26)	0.88
**Ventilation**							
No	1536	1.73 (1.25, 2.41)	–	1.45 (1.18, 1.78)	–	1.26 (1.09, 1.46)	–
Yes	5835	1.39 (1.19, 1.63)	0.24	1.23 (1.12, 1.36)	0.19	1.15 (1.07, 1.24)	0.31
**BMI category**							
Normal weight or underweight	3979	1.53 (1.26, 1.86)	–	1.32 (1.17, 1.49)	–	1.19 (1.09, 1.30)	–
Overweight or obese	3392	1.32 (1.08, 1.63)	0.31	1.20 (1.05, 1.36)	0.28	1.14 (1.03, 1.25)	0.43
**Region**							
Urban	2745	1.30 (1.05, 1.60)	–	1.19 (1.04, 1.36)	–	1.14 (1.04, 1.24)	–
Rural	4626	1.60 (1.31, 1.96)	0.16	1.34 (1.18, 1.51)	0.23	1.20 (1.10, 1.32)	0.40

The model was adjusted by age, educational level, marital status, BMI category, region, passive smoking, occupational exposure, household ventilation, biomass fuel exposure, smoking status and sex.

PM_1_, particulate matter ≤1.0µm; PM_2.5_, particulate matter ≤2.5µm; PM_10_, particulate matter ≤10µm.

a The *Z test* for the difference between the groups.

Stratified analyses suggested that the strength of associations was significantly modified by some characteristics. For instance, we observed greater associations between PMs and CB among the population exposed to biomass fuel [PM_1_ (OR: 1.75, 95%CI: 1.24∼2.47), PM_2.5_ (OR: 1.45, 95%CI: 1.18∼1.79) and PM_10_ (OR: 1.28, 95%CI: 1.10∼1.48)] than those without biomass exposure [PM_1_ (OR: 0.92, 95%CI: 0.74∼1.16), PM_2.5_ (OR: 0.98, 95%CI: 0.85∼1.12) and PM_10_ (OR: 0.99, 95%CI: 0.89∼1.09)]. We observed more pronounced associations between PM_1_ and CB among residents living in rural area [(OR: 1.39, 95%CI: 1.06∼1.80)] than those living in urban area [(OR: 0.92, 95%CI: 0.70∼1.21)] (Table S4). No notable interactions between subgroups and PM exposures were observed, probably because each variable conferred independent impact on the CRDs.

### 3.3 PAFs of CRDs attributed to PM_1_, PM_2.5_ and PM_10_

In the total population, the PAF of CRDs associated with PM_1_, PM_2.5_ and PM_10_ was 19.09% (95%CI: 12.41%∼25.02%), 18.98% (95%CI: 12.90%∼24.90%) and 21.40% (95%CI: 13.84%∼27.67%), respectively (Table 3, Fig. 1). Considering specific diseases of CRDs, the PAF associated with PMs was highest for COPD [PM_1_ (PAF: 30.53%, 95%CI: 22.83%∼37.12%), PM_2.5_ (PAF: 29.03%, 95%CI: 21.48%∼35.48%) and PM_10_ (PAF: 31.76%, 95%CI: 22.08%∼39.19%)] (Table S6, Fig. 2), followed by CB [PM_2.5_ (PAF: 9.74%, 95%CI: 0.00%∼17.77%)] (Table S7, Fig. 3) and asthma [PM_1_ (PAF: 15.62%, 95%CI: 1.81%∼26.72%), PM_2.5_ (PAF: 17.18%, 95%CI: 3.79%∼27.86%) and PM_10_ (PAF: 22.35%, 95%CI: 6.33%∼34.35%)] (Table S8, Fig. 4).

**Table 3:** **Estimated CRD burden attributable to ambient PMs exposure by different strata**.

Strata	PM_1_		PM_2.5_		PM_10_	
	Attributable cases	PAF (%)	Attributable cases	PAF (%)	Attributable cases	PAF (%)
**Total**	273 (178, 358)	19.09 (12.41, 25.02)	271 (184, 356)	18.98 (12.90, 24.90)	306 (198, 396)	21.40 (13.84, 27.67)
**Sex**						
Male	218 (134, 288)	21.10 (12.99, 27.86)	208 (130, 278)	20.16 (12.62, 26.89)	227 (128, 313)	21.95 (12.39, 30.25)
Female	70 (19, 110)	17.61 (4.69, 27.69)	77 (30, 116)	19.42 (7.44, 29.26)	89 (31, 133)	22.30 (7.79, 33.50)
**Age**						
40–50	30 (−13, 61)	14.28 (−6.38, 29.23)	36 (−4, 65)	17.56 (−2.17, 31.53)	49 (3, 81)	23.71 (1.68, 38.93)
50–60	77 (22, 119)	16.64 (4.74, 25.89)	78 (23, 119)	16.87 (5.09, 25.95)	79 (20, 129)	17.07 (4.27, 28.07)
≥60	137 (39, 213)	17.98 (5.14, 27.90)	184 (119, 242)	24.15 (15.57, 31.68)	201 (116, 269)	26.28 (15.20, 35.31)
**Smoking status**						
Never smoker	57 (0, 103)	12.06 (0.00, 21.91)	64 (9, 111)	13.55 (1.89, 23.52)	70 (7, 125)	14.90 (1.57, 26.62)
Ever smoker	239 (153, 311)	24.92 (15.89, 32.44)	226 (149, 289)	23.55 (15.48, 30.10)	250 (155, 323)	26.02 (16.17, 33.67)
**Biomass fuel exposure**						
No	122 (54, 178)	14.39 (6.34, 20.99)	123 (60, 179)	14.50 (7.05, 21.21)	144 (69, 211)	17.05 (8.19, 24.95)
Yes	166 (89, 228)	28.49 (15.31, 39.00)	168 (94, 225)	28.75 (16.11, 38.59)	158 (74, 221)	27.05 (12.64, 37.92)
**Occupational exposure**						
No	136 (72, 187)	21.02 (11.21, 28.94)	134 (75, 184)	20.73 (11.69, 28.60)	134 (63, 198)	20.79 (9.81, 30.70)
Yes	131 (56, 197)	16.68 (7.19, 25.04)	136 (64, 198)	17.27 (8.11, 25.26)	162 (80, 232)	20.65 (10.25, 29.58)
**Ventilation**						
No	73 (34, 104)	23.27 (10.79, 33.05)	76 (38, 104)	24.11 (12.21, 33.27)	81 (35, 117)	25.84 (11.04, 37.26)
Yes	201 (113, 278)	17.98 (10.17, 24.95)	199 (117, 277)	17.86 (10.44, 24.78)	223 (116, 317)	19.96 (10.43, 28.45)
**BMI category**						
Normal weight or underweight	181 (107, 245)	21.20 (12.49, 28.61)	186 (114, 248)	21.79 (13.36, 28.97)	196 (106, 271)	22.89 (12.39, 31.71)
Overweight or obese	88 (27, 141)	15.28 (4.64, 24.58)	91 (27, 141)	15.82 (4.66, 24.50)	108 (27, 167)	18.74 (4.72, 29.04)
**Region**						
Urban	64 (13, 104)	12.86 (2.65, 20.97)	68 (17, 109)	13.63 (3.41, 21.90)	92 (31, 137)	18.48 (6.16, 27.65)
Rural	231 (144, 305)	24.73 (15.41, 32.72)	222 (136, 290)	23.77 (14.60, 31.08)	205 (116, 287)	21.95 (12.43, 30.73)

PAF, population attributable fraction; PM_1_, particulate matter ≤1.0µm; PM_2.5_, particulate matter ≤2.5µm; PM_10_, particulate matter ≤10µm.

**Fig. 1:**
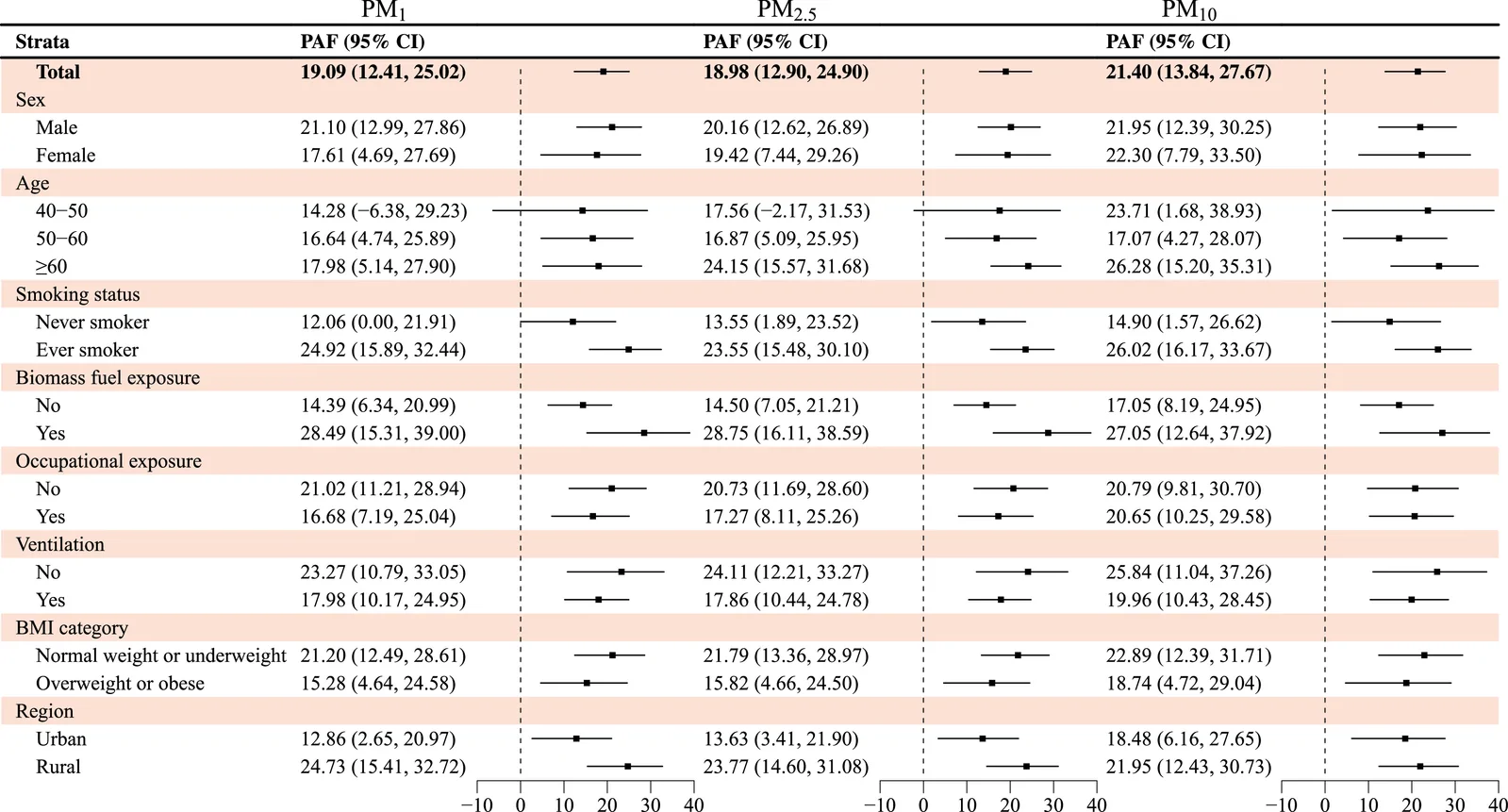
**Estimated CRDs burden attributable to ambient particulate matters by different strata**.

**Fig. 2:**
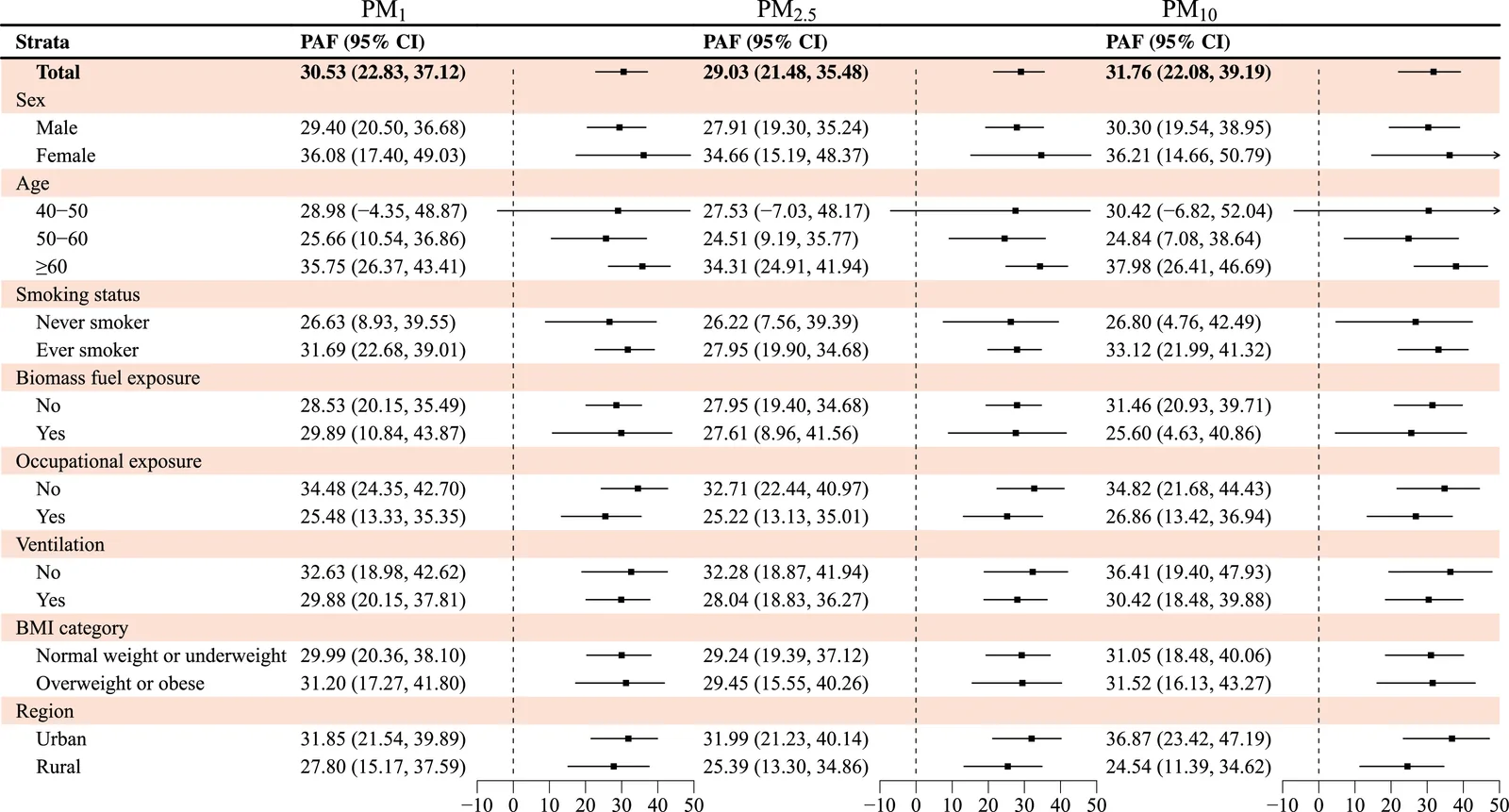
**Estimated COPD burden attributable to ambient particulate matters by different strata**.

**Fig. 3:**
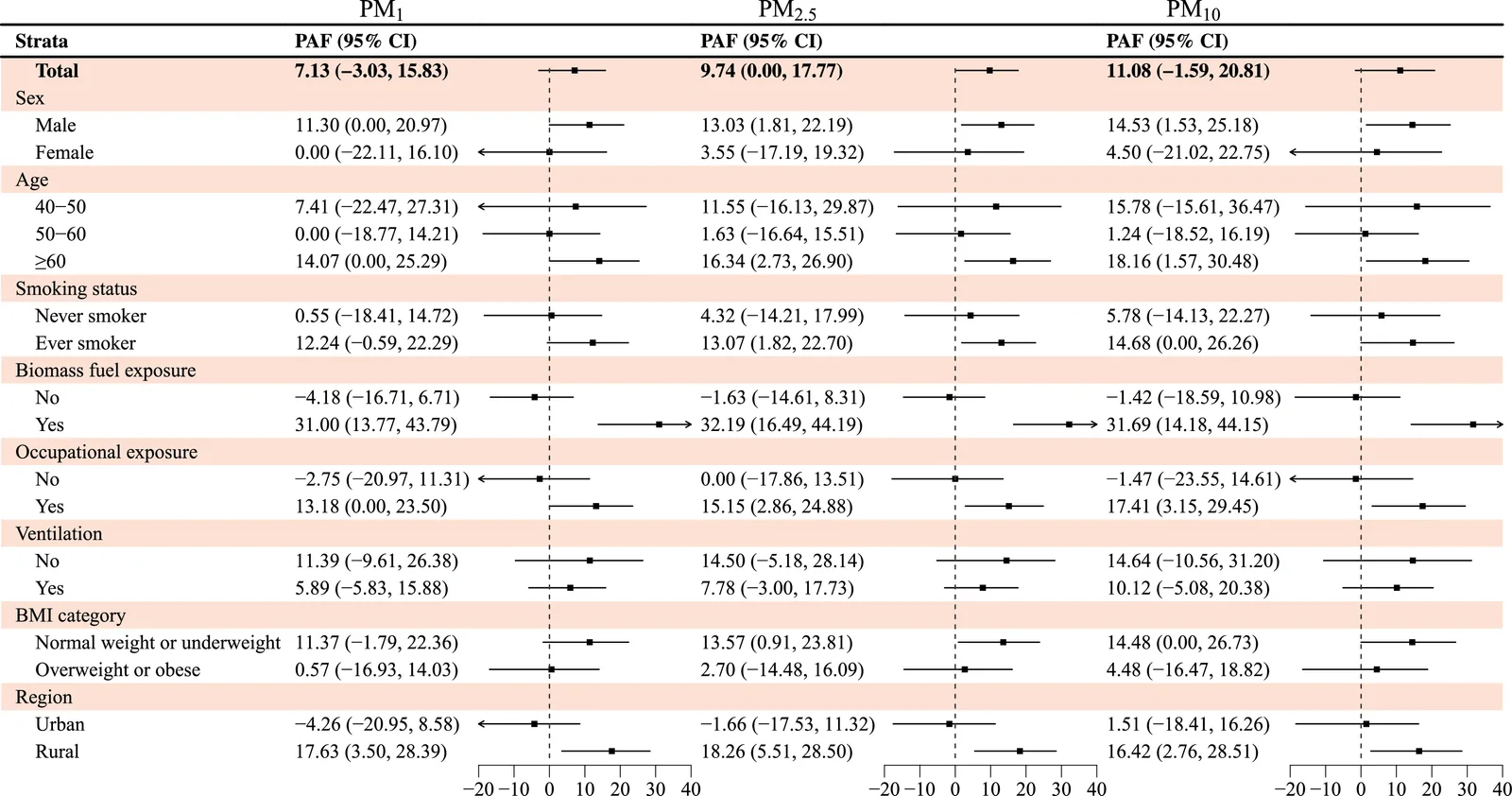
**Estimated chronic bronchitis burden attributable to particulate matters by different strata**.

**Fig. 4:**
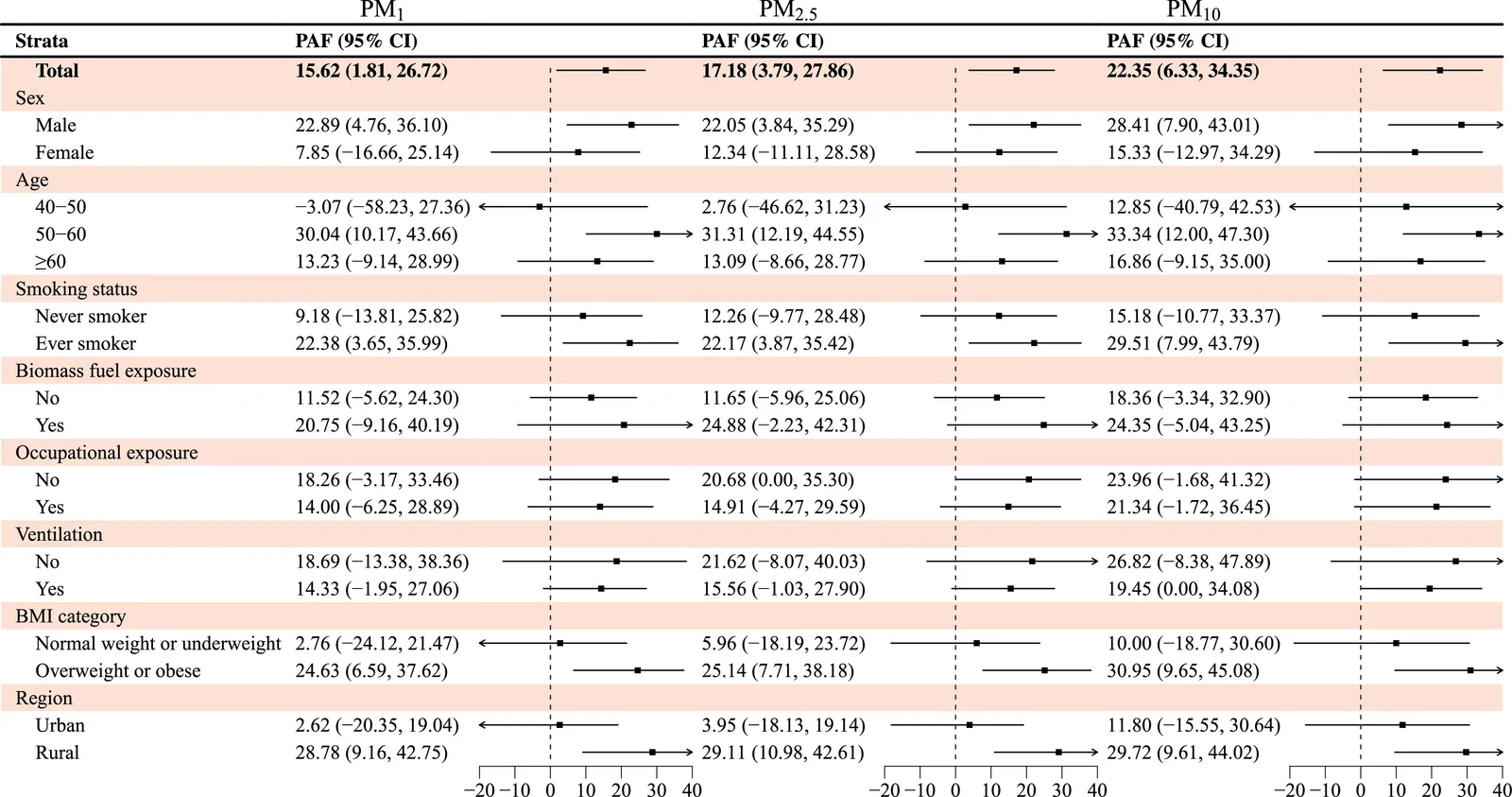
**Estimated asthma burden attributable to ambient particulate matters by different strata**.

Stratified analyses showed a major variation of PAFs among different disease components of CRDs. For instance, the PAF of CRDs ranked highest in smokers or those exposed to biomass fuel. The PAF of CRDs associated with PM_1_, PM_2.5_ and PM_10_ was (PAF: 28.49%, 95%CI: 15.31%∼39.00%), (PAF: 28.75%, 95%CI: 16.11%∼38.59%) and (PAF: 27.05%, 95%CI: 12.64%∼37.92%) among individuals exposed to biomass fuel. Details are shown in Fig. 1.

### 3.4 Sensitivity analyses

Sensitivity analyses yielded significant effect estimates even after excluding the participants with cardiovascular and other respiratory diseases. These results were not materially altered as compared with those in the total population (all P for difference test > 0.05) (Table S9). For instance, the respective OR (95%CI) for each 10 µg/m^3^ increase in PM_1_, PM_2.5_ and PM_10_ for CRDs was 1.54 (1.30, 1.83), 1.31 (1.18, 1.46) and 1.21 (1.13, 1.31) after excluding the participants with cardiovascular and other respiratory diseases.

## 4 Discussion

### 4.1 Main findings

This study has simultaneously examined the long-term associations of ambient PM exposure with CRDs and the attributable CRDs burden associated with ambient PM exposures in China. Long-term exposure to PM_1_, PM_2.5_ and PM_10_ was associated with an increased risk of CRDs. Importantly, 19.09%, 18.98% and 21.40% of CRD cases were attributable to PM_1_, PM_2.5_ and PM_10_ exposure. Furthermore, 30.53%, 29.03% and 31.76% of participants with COPD; 7.13%, 9.74% and 11.08% of participants with CB, and 15.62%, 17.18% and 22.35% of participants with asthma were attributable to PM_1_, PM_2.5_ and PM_10_ exposures. Our findings have provided the scientific evidence for estimating the CRDs burden related to PMs pollution and underlined the need for strengthening air quality regulations and pollution control measures in future policy making.

### 4.2 Interpretations

Few studies have reported the attributable risk of long-term exposure to PMs associated with the prevalence of CRDs in adults aged ≥40 years, compared with the studies focusing on short-term exposures of PM_2.5_ or PM_10_ on COPD and asthma. Overall, our findings concurred with the published studies, revealing that long-term exposure to PMs was associated with a larger proportion of patients with CRDs, including COPD and asthma [34,35]. A cross-sectional study among the elderly in six low- and middle-income countries reported that long-term exposure to PM_2.5_ might be an important risk factor for asthma [34]. Among a spatial analysis of nationwide cross-sectional study, PM_2.5_ was an important risk factor for COPD in the north, northeast, and southwest of China [35]. Additionally, some studies have indicated that long-term exposure to PMs was consistently associated with an increased risk of the incidence, mortality or hospital admission of CRDs [[36], [37], [38], [39]]. However, two studies have not detected significant positive associations of PMs with the incidence of COPD and asthma [15,40]. For instance, a prospective cohort study from north China reported no association between long-term exposure to each 10 µg/m^3^ increment in PM_2.5_ concentration and the incidence of asthma and COPD [15], although positive effects on CRDs and CB were identified. The BIGEPI study did not demonstrate a significant association between long-tern exposure to PM_2.5_ and PM_10_ and the risk of CRDs in Italy [40]. The principal findings among these studies cannot be compared directly due to the notable differences in the residential regions, study population, ventilator conditions, PMs concentration and components. The different magnitude of associations among studies have rendered the associations of long-term exposure to PMs with CRDs and the individual components difficult to be fully ascertained. Moreover, we did not find significant associations of long-term exposure to PM_1_ with CB in south China. For stratified analyses, we noted stronger associations between PMs and the risk of CRDs and CB in participants exposed to biomass fuel or having lived in rural areas. Stronger associations were observed in participants having lived in rural areas, possibly because the participants were more likely to have lower education levels, have smoked, have used unclean cooking fuel and have poorer lung function [17]. Use of biomass fuel could also increase the risk of CRDs through similar mechanisms of atmospheric air pollution [[41], [42], [43], [44], [45]], synergizing the effects of ambient PMs pollutants.

The PAF of PMs attributable to CRDs was substantial. The PAF of PM_2.5_ attributable to COPD was higher (29.03%) than that in the SAGE study (13.79%) [14]. We have performed bronchial dilation test, the gold standard for COPD diagnosis, while COPD was self-reported in the SAGE study, thus accounting for the lower under-reporting rate and higher PAF of PM_2.5_ to COPD in our study.

### 4.3 Mechanism of the association between PMs and CRDs

First, patients with CRDs exposed to air pollution had abnormal regulation of proteins involved in oxidative stress, cell metabolism, inflammatory response, and actin dynamics [[41], [42], [43], [44], [45]]. Second, PMs induced defective cilia clearance of PM from the lungs and airways [46]. Third, long-term exposure to ambient PMs might regulate lung damage through DNA methylation [47]. Moreover, PMs could elicit pulmonary microvascular injury by accelerating the apoptosis of vascular endothelial cells, increasing vascular permeability, and reducing angiogenesis [48].

### 4.4 Limitations and strengths

First, this was a population-based cross-sectional study, so it could not establish causality between PM exposure and CRDs. Second, food intake, physical activity, vaccinations and indoor environmental pollutants (molds, household fuels, and allergens) were not collected, which might have confounded the association between PM exposure and CRDs [49]. Third, we employed a simplified definition of COPD, which could lead to misclassification and overestimation of COPD prevalence, potentially influencing the association with PM exposure. Fourth, our study might have been biased by the average pollutant exposure levels for participants who had lived at the site for less than 722 days. The global lack of high-quality PMs estimates at finer spatiotemporal scales precludes the more comparative analysis using various exposure datasets. Finally, our study did not collect sufficient data to analyze the time spent by participants indoors versus outdoors. In the next round of epidemiological surveys, participants would be invited to wear personal air pollutant exposure monitors to obtain more accurate individual-level exposure data.

Despite the limitations, our study has included a relatively large sample of middle-aged and elderly population, allowing for a robust analysis to detect the associations for PM with CRDs based on a comprehensive panel of covariates for adjustment. Second, we have estimated the residential concentrations of PM_s_ at a spatial resolution of about 1 km^2^, a reasonably fine precision of exposure modelling that has been validated in previous studies [[30], [31], [32]] to safeguard the accuracy of the exposure assessment. Additionally, our association analyses were based on sensitivity analyses, excluding the participants with known cardiovascular and other respiratory diseases, with the overall results not being altered materially.

### 4.5 Implications

About 19.09%, 18.98% and 21.40% of CRD cases could be attributable to PM_1_, PM_2.5_ and PM_10__,_ exposures in South China. Considering air pollution exposure is ubiquitous for each individual and that the prevalence of CRDs (especially COPD) is high in China, the effects of air pollution on CRDs indicate a major public health burden. Approximately 7,000,376 incident Chinese cases aged 55 years or greater with CRDs were documented in 2019 [50]. The corresponding figures would add up to 1,336,371, 1,328,671 and 1,498,080 cases with CRDs elicited by the exposure to PM_1_, PM_2.5_ and PM_10_. The significantly higher PAFs of CRDs due to long-term exposure to ambient PMs in South China underscores the urgency of incorporating environmental governance and PM pollution control into public health strategies. These measures are essential to mitigating the substantial burden of CRDs on regional health.

Environmental governance is crucial for mitigating the adverse health impacts of air pollution. However, clinicians and public health experts also play a vital role in providing short-term protection for high-risk populations. This can be achieved by promoting protective knowledge and interventions in communities and advising high-risk groups, including children, the elderly, and individuals with cardiovascular or respiratory diseases, to reduce outdoor activities during severe pollution episodes and to use high-efficiency air purifiers and portable monitors to mitigate indoor pollution. At the individual level, promoting the use of protective masks (e.g., N95 masks), encouraging intake of antioxidant-rich foods or vitamin C and E supplements to reduce oxidative stress, and improving indoor ventilation, reducing the use of solid fuels for cooking, and avoiding second-hand smoke exposure are all effective measures to lower the health risks associated with air pollution [4].

## 5 Conclusion

Long-term exposures to ambient PMs are associated with a higher proportion of patients with CRDs. PM pollution is a significant and preventable risk factor for CRDs. Effective interventions are needed to control PM pollution and reduce the attributable burden of CRDs.

## Data availability

The datasets generated and analyzed during the current study are not publicly available but are available from the corresponding author on reasonable request.

## CRediT authorship contribution statement

**Wei-jie Guan:** Conceptualization, Formal analysis, Funding acquisition, Methodology, Project administration, Resources, Supervision, Validation, Writing – original draft, Writing – review & editing. **Shu-jun Guo:** Conceptualization, Data curation, Formal analysis, Methodology, Software, Visualization, Writing – review & editing. **Li-lian Zeng:** Conceptualization, Methodology, Software, Validation, Writing – original draft. **Jing Wei:** Resources, Validation, Writing – review & editing. **De-jian Zhao:** Investigation, Writing – review & editing. **Rui-lin Meng:** Investigation, Project administration, Resources, Supervision, Writing – review & editing. **Yan-jun Xu:** Resources, Writing – review & editing. **Yun-hong Lv:** Investigation, Resources, Supervision, Writing – review & editing. **Chuan Li:** Investigation, Writing – review & editing. **Hong-ye Zhou:** Conceptualization, Data curation, Methodology, Software. **Jia-hui He:** Data curation, Methodology. **Li-feng Lin:** Conceptualization, Investigation, Project administration, Resources, Supervision, Writing – review & editing. **Xue-yan Zheng:** Conceptualization, Data curation, Formal analysis, Funding acquisition, Investigation, Methodology, Project administration, Resources, Software, Supervision, Validation, Writing – original draft, Writing – review & editing.

## Declaration of competing interest

The authors declare that they have no conflicts of interest in this work.

## Appendix Supplementary materials

Image, application 1

Image, application 2
