# Supplementary material for: Association between chronic respiratory diseases and the attributable risk of long-term exposure to atmospheric particulate matters among middle-aged and elderly Chinese population
Source: Fundam Res. 2025 Apr 29;6(5):3408–16. doi: 10.1016/j.fmre.2025.04.013 (PMC13644410; doi:10.1016/j.fmre.2025.04.013)
Supplement: Supplementary file 2 [file mmc1.doc]

**Online supplement**

**Association between chronic respiratory diseases and the attributable risk of long-term exposure to atmospheric particulate matters among middle-aged and elderly Chinese population**

**Figure legends**

**Figure S1. Exposure-response curves of long-term exposure to PMs with CRDs in south China.**

**Method**

**Sampling strategy**

The details of sampling have been published previously. This study included adults aged ≥40 years who participated in the Chinese National COPD Surveillance in Guangdong Province during 2014–2015 and 2019–2020. The project used a multistage stratified cluster random sampling method. The country was divided into 12 strata based on region (eastern, central, and western), level of urbanization (high and low), and gender (male and female). Six representative survey sites (cities, counties, or districts) were selected from the 21 prefecture-level cities in Guangdong: Panyu District of Guangzhou, Doumen District of Zhuhai, Yingde City of Qingyuan, Taishan City of Jiangmen, Wengyuan County of Shaoguan, and Luoding City of Yunfu. In each monitoring site, three townships/streets were selected using Probability Proportionate to Size (PPS) sampling, proportional to the population size. In each selected township/street, two administrative villages/neighborhood committees were selected using the PPS method. In each selected administrative village/neighborhood committee, one village group (natural village)/resident group was selected using cluster random sampling, with each village group (natural village)/resident group including at least 150 households. In each selected village group (natural village)/resident group, 100 households containing residents aged 40 years or older were randomly selected for the survey. If the selected household was ineligible or unavailable for the survey, it would be replaced, with the replacement rate controlled at less than 10%.

All analyses have been adjusted for age, educational level, marital status, BMI category, residential region, passive smoking, occupational exposure, household ventilation, biomass fuel exposure, smoking status and sex.

CRD: chronic respiratory diseases; COPD: chronic obstructive pulmonary diseases; CB: chronic bronchitis; OR: odd ratio; PM_1_: Particulate matter with an aerodynamic diameter of 1.0 μm or less; PM_2.5_: particulate matter to aerodynamic diameters≤ 2.5 μm; PM_10_: particulate matter to aerodynamic diameters≤ 10 μm.

**Table S1 Main characteristics between the included and the total population.**

| **Characteristics** | **Total**  **(No.=7507^a^)** | **Included**  **(No.=7371)** | ***P value*** |
| --- | --- | --- | --- |
| **Social demographic indices (SDI)** |  |  |  |
| **Sex, n (%)** |  |  | 0.95 |
| Male | 3837 (51.11) | 3764 (51.06) |  |
| Female | 3670 (48.89) | 3607 (48.94) |  |
| **Age (year), mean (SD)** | 57.35 (9.33) | 57.26 (9.26) | 0.74 |
| **Educational level, n (%)** |  |  | 0.98 |
| None + primary school education | 4321 (57.56) | 4231 (57.40) |  |
| Middle school education | 3033 (40.40) | 2989 (40.55) |  |
| University education or higher | 153 (2.04) | 151 (2.05) |  |
| **Marriage status, n (%)** |  |  | 0.96 |
| Unmarried | 99 (1.32) | 94 (1.28) |  |
| Married | 6836 (91.06) | 6720 (91.17) |  |
| Widowed or divorced | 572 (7.62) | 557 (7.56) |  |
| **Region of residence, n (%)** |  |  | 1.00 |
| Urban | 2796 (37.25) | 2745 (37.24) |  |
| Rural | 4711 (62.75) | 4626 (62.76) |  |
| **Household air pollution exposure, n (%)** |  |  |  |
| **Household ventilation** |  |  | 0.88 |
| No | 1572 (20.94) | 1536 (20.84) |  |
| Yes | 5935 (79.06) | 5835 (79.16) |  |
| **Passive smoking** |  |  | 0.81 |
| No | 3244 (43.21) | 3171 (43.02) |  |
| Yes | 4263 (56.79) | 4200 (56.98) |  |
| **Biomass exposure** |  |  | 0.98 |
| No | 4717 (62.83) | 4633 (62.85) |  |
| Yes | 2790 (37.17) | 2738 (37.15) |  |
| **Smoking status** |  |  | 0.99 |
| Never smoker | 4266 (56.83) | 4188 (56.82) |  |
| Ever smoker | 3241 (43.17) | 3183 (43.18) |  |
| **Occupational exposure** |  |  | 0.64 |
| No | 3792 (50.51) | 3695 (50.13) |  |
| Yes | 3715 (49.49) | 3676 (49.87) |  |
| **Anthropometry** |  |  |  |
| **BMI** **category, n (%)** |  |  |  |
| Normal weight or underweight | 3979 (53.98) | 3979 (53.98) | -^b^ |
| Overweight or obese | 3392 (46.02) | 3392 (46.02) | - |
| **Ambient air pollutants exposure** |  |  |  |
| PM_1_ | 20.82 (4.33) | 20.85 (4.34) | 0.74 |
| PM_2.5_ | 34.88 (6.96) | 34.92 (6.97) | 0.72 |
| PM_10_ | 54.30 (9.87) | 54.35 (9.89) | 0.74 |
| **Chronic respiratory diseases^c^** |  |  |  |
| **Chronic Bronchitis** |  |  | 0.82 |
| Yes | 753 (10.03) | 731 (9.92) |  |
| No | 6754 (89.97) | 6640 (90.08) |  |
| **Asthma** |  |  | 0.80 |
| Yes | 325 (4.33) | 313 (4.25) |  |
| No | 7182 (95.67) | 7058 (95.75) |  |

**^a^:** Regarding that information on questionnaire are major items for analyzing basic characteristics, and that three participants did not answer symptom related questions, we included 7507 participants as the total population for comparison.

^b^: Regarding that 136 did not have height data, the number of participants for the total and included group were the same.

^c^: We only considered the comparison of participants with chronic bronchitis and asthma, because excluded participants could not diagnose with COPD and CRDs.

*P*: The t-tests were performed to determine the association between the continuous variables, and when indicated, appropriate transformations were made. Contingency tables and chi-squared tests were employed for categorical variables to compare the percentages. *P* values< 0.05 were considered as statistically significant.

**Table S2** **Summary statistics and Spearman’s correlation of the 722-day mean air pollutants.**

|  | **Summary statistics** | | | | |  | **Spearman’s correlation coefficients** | | | | | | |
| --- | --- | --- | --- | --- | --- | --- | --- | --- | --- | --- | --- | --- | --- |
|  | **Mean (SD)** | **Median** | **Minimum** | **Maximum** | **IQR** |  | **PM_1_** | **PM_2.5_** | **PM_10_** | **SO_2_** | **CO** | **NO_2_** | **O_3_** |
| **PM_1_ (μg/m^3^)** | 20.85 (4.34) | 18.95 | 14.96 | 31.54 | 6.72 |  | 1.00 | 0.99* | 0.96* | 0.87* | 0.80* | 0.40* | -0.29* |
| **PM_2.5_ (μg/m^3^)** | 34.92 (6.97) | 32.74 | 24.13 | 48.74 | 10.81 |  | 0.99* | 1.00 | 0.97* | 0.87* | 0.80* | 0.41* | -0.28* |
| **PM_10_ (μg/m^3^)** | 54.35 (9.89) | 52.90 | 39.50 | 73.05 | 16.39 |  | 0.96* | 0.97* | 1.00 | 0.83* | 0.78* | 0.48* | -0.19* |
| **SO_2_ (μg/m^3^)** | 15.94 (6.96) | 13.27 | 6.19 | 31.31 | 12.49 |  | 0.87* | 0.87* | 0.83* | 1.00 | 0.91* | 0.05* | -0.60* |
| **CO (mg/m^3^)** | 1.01 (0.19) | 1.01 | 0.72 | 1.40 | 0.31 |  | 0.80* | 0.80* | 0.78* | 0.91* | 1.00 | 0.04* | -0.57* |
| **NO_2_(μg/m^3^)** | 29.09 (8.03) | 27.56 | 19.61 | 47.29 | 8.64 |  | 0.40* | 0.41* | 0.48* | 0.05* | 0.04* | 1.00 | 0.50* |

PM_1_: particulate matter≤ 1.0µm; PM_2.5_: particulate matter≤ 2.5µm; PM_10_: particulate matter≤ 10µm; SO_2_: sulfur dioxide; CO: carbon monoxide;

Note: Spearman correlation coefficients, ^*^: *P* < 0.05

**Table S3. Adjusted OR and 95% CI for COPD associated with each 10 μg/m^3^ increase in PMs by stratification.**

| **Stratifications** | **No. of Participants** | **COPD** | | | | | |
| --- | --- | --- | --- | --- | --- | --- | --- |
|  |  | **PM_1_** | | **PM_2.5_** | | **PM_10_** | |
|  |  | **OR**  **(95% CI)** | ***P* for difference test****^*^** | **OR**  **(95% CI)** | ***P* for difference test^*^** | **OR**  **(95% CI)** | ***P* for difference test^*^** |
| **Total** | 7371 | 1.85 (1.53, 2.24) | - | 1.45 (1.29, 1.63) | - | 1.28 (1.17, 1.39) | - |
| **Sex** |  |  |  |  |  |  |  |
| Male | 3764 | 1.83 (1.47, 2.27) | - | 1.44 (1.26, 1.65) | - | 1.27 (1.15, 1.40) | - |
| Female | 3607 | 1.99 (1.32, 3.00) | 0.70 | 1.50 (1.16, 1.95) | 0.79 | 1.33 (1.10, 1.61) | 0.65 |
| **Age** |  |  |  |  |  |  |  |
| 40-50 | 1776 | 1.71 (0.94, 3.13) | - | 1.37 (0.94, 2.00) | - | 1.27 (0.96, 1.68) | - |
| 50-60 | 2747 | 1.71 (1.21, 2.41) | 1.00 | 1.38 (1.11, 1.71) | 1.00 | 1.22 (1.05, 1.43) | 0.80 |
| ≥60 | 2844 | 2.12 (1.65, 2.73) | 0.53 | 1.58 (1.35, 1.85) | 0.50 | 1.36 (1.21, 1.52) | 0.69 |
| **Smoking status** |  |  |  |  |  |  |  |
| Never smoker | 4188 | 1.68 (1.16, 2.44) | - | 1.37 (1.08, 1.73) | - | 1.22 (1.03, 1.45) | - |
| Ever smoker | 3183 | 1.92 (1.53, 2.40) | 0.56 | 1.48 (1.29, 1.70) | 0.61 | 1.30 (1.17, 1.43) | 0.56 |
| **Biomass fuel exposure** |  |  |  |  |  |  |  |
| No | 4633 | 1.88 (1.50, 2.36) | - | 1.48 (1.28, 1.70) | - | 1.30 (1.17, 1.44) | - |
| Yes | 2738 | 1.70 (1.18, 2.45) | 0.66 | 1.36 (1.09, 1.70) | 0.54 | 1.21 (1.03, 1.42) | 0.46 |
| **Occupational exposure** |  |  |  |  |  |  |  |
| No | 3695 | 2.10 (1.60, 2.77) | - | 1.57 (1.32, 1.87) | - | 1.33 (1.17, 1.50) | - |
| Yes | 3676 | 1.64 (1.26, 2.15) | 0.23 | 1.35 (1.15, 1.59) | 0.21 | 1.24 (1.10, 1.39) | 0.41 |
| **Ventilation** |  |  |  |  |  |  |  |
| No | 1536 | 2.26 (1.50, 3.42) | - | 1.69 (1.30, 2.19) | - | 1.42 (1.17, 1.71) | - |
| Yes | 5835 | 1.77 (1.42, 2.20) | 0.29 | 1.40 (1.23, 1.61) | 0.20 | 1.25 (1.13, 1.38) | 0.24 |
| **BMI category** |  |  |  |  |  |  |  |
| Normal weight or underweight | 3979 | 1.87 (1.47, 2.40) | - | 1.47 (1.26, 1.71) | - | 1.28 (1.14, 1.42) | - |
| Overweight or obese | 3392 | 1.83 (1.34, 2.50) | 0.92 | 1.43 (1.18, 1.74) | 0.81 | 1.29 (1.12, 1.49) | 0.91 |
| **Region** |  |  |  |  |  |  |  |
| Urban | 2745 | 2.00 (1.51, 2.66) | - | 1.55 (1.29, 1.86) | - | 1.33 (1.17, 1.52) | - |
| Rural | 4626 | 1.71 (1.30, 2.23) | 0.44 | 1.37 (1.16, 1.61) | 0.28 | 1.23 (1.09, 1.38) | 0.33 |

The model was adjusted by age, educational level, marital status, BMI category, region, passive smoking, occupational exposure, household ventilation, biomass fuel exposure, smoking status and sex.

PM_1_: particulate matter≤ 1.0µm; PM_2.5_: particulate matter≤ 2.5µm; PM_10_: particulate matter≤ 10µm.

**^*^**: the Z test for the difference between groups.

**Table S4. Adjusted OR and 95% CI for chronic bronchitis associated with each 10 μg/m^3^ increase in PMs by stratification.**

| **Stratifications** | **No. of Participants** | **Chronic Bronchitis** | | | | | |
| --- | --- | --- | --- | --- | --- | --- | --- |
|  |  | **PM_1_** | | **PM_2.5_** | | **PM_10_** | |
|  |  | **OR**  **(95% CI)** | ***P* for difference test****^*^** | **OR**  **(95% CI)** | ***P* for difference test^*^** | **OR**  **(95% CI)** | ***P* for difference test^*^** |
| **Total** | 7371 | 1.14 (0.95, 1.37) | - | 1.12 (1.00, 1.25) | - | 1.08 (0.99, 1.17) | - |
| **Sex** |  |  |  |  |  |  |  |
| Male | 3764 | 1.24 (1.00, 1.55) | - | 1.17 (1.02, 1.34) | - | 1.11 (1.01, 1.22) | - |
| Female | 3607 | 1.00 (0.72, 1.38) | 0.28 | 1.04 (0.85, 1.28) | 0.33 | 1.03 (0.89, 1.19) | 0.42 |
| **Age** |  |  |  |  |  |  |  |
| 40-50 | 1776 | 1.13 (0.74, 1.72) | - | 1.13 (0.87, 1.46) | - | 1.11 (0.92, 1.34) | - |
| 50-60 | 2747 | 1.00 (0.73, 1.37) | 0.65 | 1.02 (0.84, 1.24) | 0.54 | 1.01 (0.88, 1.16) | 0.46 |
| ≥60 | 2844 | 1.31 (1.00, 1.72) | 0.55 | 1.22 (1.03, 1.44) | 0.61 | 1.14 (1.01, 1.28) | 0.80 |
| **Smoking status** |  |  |  |  |  |  |  |
| Never smoker | 4188 | 1.01 (0.75, 1.35) | - | 1.05 (0.87, 1.26) | - | 1.04 (0.92, 1.19) | - |
| Ever smoker | 3183 | 1.26 (0.99, 1.59) | 0.25 | 1.17 (1.02, 1.35) | 0.33 | 1.11 (1.00, 1.23) | 0.49 |
| **Biomass fuel exposure** |  |  |  |  |  |  |  |
| No | 4633 | 0.92 (0.74, 1.16) | - | 0.98 (0.85, 1.12) | - | 0.99 (0.89, 1.09) | - |
| Yes | 2738 | 1.75 (1.24, 2.47) | 0.002^**^ | 1.45 (1.18, 1.79) | 0.003^**^ | 1.28 (1.10, 1.48) | 0.006^**^ |
| **Occupational exposure** |  |  |  |  |  |  |  |
| No | 3695 | 0.95 (0.71, 1.27) | - | 1.00 (0.83, 1.20) | - | 0.99 (0.87, 1.12) | - |
| Yes | 3676 | 1.27 (1.00, 1.60) | 0.13 | 1.19 (1.03, 1.37) | 0.14 | 1.13 (1.02, 1.26) | 0.10 |
| **Ventilation** |  |  |  |  |  |  |  |
| No | 1536 | 1.28 (0.84, 1.96) | - | 1.23 (0.94, 1.60) | - | 1.13 (0.93, 1.36) | - |
| Yes | 5835 | 1.11 (0.91, 1.36) | 0.56 | 1.09 (0.97, 1.24) | 0.47 | 1.07 (0.97, 1.16) | 0.59 |
| **BMI category** |  |  |  |  |  |  |  |
| Normal weight or underweight | 3979 | 1.24 (0.97, 1.60) | - | 1.18 (1.01, 1.38) | - | 1.11 (1.00, 1.24) | - |
| Overweight or obese | 3392 | 1.01 (0.77, 1.32) | 0.27 | 1.03 (0.87, 1.22) | 0.22 | 1.03 (0.91, 1.15) | 0.35 |
| **Region** |  |  |  |  |  |  |  |
| Urban | 2745 | 0.92 (0.70, 1.21) | - | 0.98 (0.83, 1.17) | - | 1.01 (0.90, 1.13) | - |
| Rural | 4626 | 1.39 (1.06, 1.80) | 0.03^**^ | 1.24 (1.06, 1.45) | 0.06 | 1.14 (1.02, 1.29) | 0.16 |

The model was adjusted by age, educational level, marital status, BMI category, region, passive smoking, occupational exposure, household ventilation, biomass fuel exposure, smoking status and sex.

PM_1_: particulate matter≤1.0µm; PM_2.5_: particulate matter≤2.5µm; PM_10_: particulate matter≤ 10µm.

**^*^**: the Z test for the difference between groups.

**^**^**: the *P* value< 0.05.

**Table S5. Adjusted OR and 95% CI for asthma associated with each 10 μg/m^3^ increase in PMs by stratification.**

| **Stratifications** | **No. of Participants** | **Asthma** | | | | | |
| --- | --- | --- | --- | --- | --- | --- | --- |
|  |  | **PM_1_** | | **PM_2.5_** | | **PM_10_** | |
|  |  | **OR**  **(95% CI)** | ***P* for difference test^*^** | **OR**  **(95% CI)** | ***P* for difference test^*^** | **OR**  **(95% CI)** | ***P* for difference test^*^** |
| **Total** | 7371 | 1.33 (1.03, 1.72) | - | 1.22 (1.04, 1.43) | - | 1.17 (1.04, 1.31) | - |
| **Sex** |  |  |  |  |  |  |  |
| Male | 3764 | 1.54 (1.08, 2.19) | - | 1.30 (1.04, 1.62) | - | 1.23 (1.05, 1.44) | - |
| Female | 3607 | 1.15 (0.78, 1.69) | 0.28 | 1.15 (0.90, 1.46) | 0.46 | 1.11 (0.93, 1.32) | 0.36 |
| **Age** |  |  |  |  |  |  |  |
| 40-50 | 1776 | 0.95 (0.49, 1.81) | - | 1.03 (0.69, 1.54) | - | 1.09 (0.82, 1.46) | - |
| 50-60 | 2747 | 1.86 (1.19, 2.91) | 0.09 | 1.50 (1.14, 1.98) | 0.12 | 1.34 (1.09, 1.64) | 0.27 |
| ≥60 | 2844 | 1.27 (0.87, 1.84) | 0.43 | 1.16 (0.92, 1.46) | 0.61 | 1.12 (0.95, 1.32) | 0.91 |
| **Smoking status** |  |  |  |  |  |  |  |
| Never smoker | 4188 | 1.18 (0.81, 1.72) | - | 1.15 (0.91, 1.46) | - | 1.11 (0.94, 1.31) | - |
| Ever smoker | 3183 | 1.52 (1.06, 2.18) | 0.34 | 1.30 (1.04, 1.62) | 0.46 | 1.24 (1.05, 1.45) | 0.36 |
| **Biomass fuel exposure** |  |  |  |  |  |  |  |
| No | 4633 | 1.25 (0.91, 1.71) | - | 1.15 (0.94, 1.41) | - | 1.14 (0.98, 1.31) | - |
| Yes | 2738 | 1.43 (0.88, 2.31) | 0.64 | 1.32 (0.98, 1.76) | 0.47 | 1.20 (0.97, 1.48) | 0.70 |
| **Occupational exposure** |  |  |  |  |  |  |  |
| No | 3695 | 1.42 (0.95, 2.12) | - | 1.29 (1.00, 1.66) | - | 1.19 (0.99, 1.43) | - |
| Yes | 3676 | 1.28 (0.91, 1.80) | 0.71 | 1.18 (0.96, 1.46) | 0.64 | 1.16 (0.99, 1.34) | 0.74 |
| **Ventilation** |  |  |  |  |  |  |  |
| No | 1536 | 1.46 (0.81, 2.63) | - | 1.33 (0.92, 1.92) | - | 1.24 (0.95, 1.63) | - |
| Yes | 5835 | 1.29 (0.97, 1.72) | 0.70 | 1.19 (0.99, 1.42) | 0.57 | 1.14 (1.00, 1.30) | 0.57 |
| **BMI category** |  |  |  |  |  |  |  |
| Normal weight or underweight | 3979 | 1.05 (0.70, 1.56) | - | 1.07 (0.84, 1.37) | - | 1.07 (0.9, 1.28) | - |
| Overweight or obese | 3392 | 1.58 (1.11, 2.24) | 0.13 | 1.34 (1.08, 1.67) | 0.18 | 1.25 (1.06, 1.46) | 0.21 |
| **Region** |  |  |  |  |  |  |  |
| Urban | 2745 | 1.05 (0.73, 1.52) | - | 1.05 (0.83, 1.32) | - | 1.08 (0.92, 1.27) | - |
| Rural | 4626 | 1.73 (1.16, 2.57) | 0.07 | 1.42 (1.12, 1.81) | 0.08 | 1.28 (1.07, 1.53) | 0.16 |

The model was adjusted by age, educational level, marital status, BMI category, region, passive smoking, occupational exposure, household ventilation, biomass fuel exposure, smoking status and sex.

PM_1_: particulate matter≤1.0µm; PM_2.5_: particulate matter≤2.5µm; PM_10_: particulate matter≤ 10µm.

**^*^**: the Z test for the difference between groups.

**Table S6. The population-attributable fraction of chronic obstructive pulmonary diseases attributable to PMs by different strata**

| **Strata** | **PM_1_** | | **PM_2.5_** | | **PM_10_** | |
| --- | --- | --- | --- | --- | --- | --- |
|  | **Attributable cases** | **PAF (%)** | **Attributable cases** | **PAF (%)** | **Attributable cases** | **PAF (%)** |
| **Total** | 225 (168, 273) | 30.53 (22.83, 37.12) | 214 (158, 261) | 29.03 (21.48, 35.48) | 234 (163, 288) | 31.76 (22.08, 39.19) |
| **Sex** |  |  |  |  |  |  |
| Male | 179 (125, 224) | 29.40 (20.50, 36.68) | 170 (118, 215) | 27.91 (19.30, 35.24) | 185 (119, 238) | 30.30 (19.54, 38.95) |
| Female | 45 (22, 62) | 36.08 (17.40, 49.03) | 44 (19, 61) | 34.66 (15.19, 48.37) | 46 (18, 64) | 36.21 (14.66, 50.79) |
| **Age** |  |  |  |  |  |  |
| 40-50 | 17 (-3, 29) | 28.98 (-4.35, 48.87) | 17 (-4, 29) | 27.53 (-7.03, 48.17) | 18 (-4, 31) | 30.42 (-6.82, 52.04) |
| 50-60 | 53 (22, 76) | 25.66 (10.54, 36.86) | 51 (19, 74) | 24.51 (9.19, 35.77) | 51 (15, 80) | 24.84 (7.08, 38.64) |
| ≥60 | 168 (124, 204) | 35.75 (26.37, 43.41) | 161 (117, 197) | 34.31 (24.91, 41.94) | 178 (124, 219) | 37.98 (26.41, 46.69) |
| **Smoking status** |  |  |  |  |  |  |
| Never smoker | 42 (14, 63) | 26.63 (8.93, 39.55) | 42 (12, 63) | 26.22 (7.56, 39.39) | 43 (8, 68) | 26.80 (4.76, 42.49) |
| Ever smoker | 183 (131, 225) | 31.69 (22.68, 39.01) | 123 (88, 153) | 27.95 (19.90, 34.68) | 191 (127, 238) | 33.12 (21.99, 41.32) |
| **Biomass fuel exposure** |  |  |  |  |  |  |
| No | 126 (89, 157) | 28.53 (20.15, 35.49) | 123 (86, 153) | 27.95 (19.40, 34.68) | 139 (92, 175) | 31.46 (20.93, 39.71) |
| Yes | 88 (32, 129) | 29.89 (10.84, 43.87) | 81 (26, 123) | 27.61 (8.96, 41.56) | 76 (14, 121) | 25.60 (4.63, 40.86) |
| **Occupational exposure** |  |  |  |  |  |  |
| No | 127 (90, 157) | 34.48 (24.35, 42.70) | 120 (83, 151) | 32.71 (22.44, 40.97) | 128 (80, 164) | 34.82 (21.68, 44.43) |
| Yes | 94 (49, 130) | 25.48 (13.33, 35.35) | 93 (48, 129) | 25.22 (13.13, 35.01) | 99 (49, 136) | 26.86 (13.42, 36.94) |
| **Ventilation** |  |  |  |  |  |  |
| No | 62 (36, 81) | 32.63 (18.98, 42.62) | 62 (36, 80) | 32.28 (18.87, 41.94) | 70 (37, 92) | 36.41 (19.40, 47.93) |
| Yes | 163 (110, 206) | 29.88 (20.15, 37.81) | 153 (103, 198) | 28.04 (18.83, 36.27) | 166 (101, 217) | 30.42 (18.48, 39.88) |
| **BMI category** |  |  |  |  |  |  |
| Normal weight or underweight | 151 (103, 192) | 29.99 (20.36, 38.10) | 147 (98, 187) | 29.24 (19.39, 37.12) | 157 (93, 202) | 31.05 (18.48, 40.06) |
| Overweight or obese | 72 (40, 97) | 31.20 (17.27, 41.80) | 68 (36, 93) | 29.45 (15.55, 40.26) | 73 (37, 100) | 31.52 (16.13, 43.27) |
| **Region** |  |  |  |  |  |  |
| Urban | 75 (51, 95) | 31.85 (21.54, 39.89) | 76 (50, 95) | 31.99 (21.23, 40.14) | 87 (56, 112) | 36.87 (23.42, 47.19) |
| Rural | 139 (76, 188) | 27.80 (15.17, 37.59) | 127 (66, 174) | 25.39 (13.30, 34.86) | 122 (57, 173) | 24.54 (11.39, 34.62) |

PAF: population attributable fraction; PM_1_: particulate matter ≤1.0µm; PM_2.5_: particulate matter ≤2.5µm; PM_10_: particulate matter ≤ 10µm.

**Table S7. The population-attributable fraction of chronic bronchitis attributable to PMs by different strata**

| **Strata** | **PM_1_** | | **PM_2.5_** | | **PM_10_** | |
| --- | --- | --- | --- | --- | --- | --- |
|  | **Attributable cases** | **PAF (%)** | **Attributable cases** | **PAF (%)** | **Attributable cases** | **PAF (%)** |
| **Total** | 52 (-22, 116) | 7.13 (-3.03, 15.83) | 71 (0, 130) | 9.74 (0.00, 17.77) | 81 (-12, 152) | 11.08 (-1.59, 20.81) |
| **Sex** |  |  |  |  |  |  |
| Male | 59 (0, 109) | 11.30 (0.00, 20.97) | 68 (9, 116) | 13.03 (1.81, 22.19) | 76 (8, 131) | 14.53 (1.53, 25.18) |
| Female | 0 (-46, 34) | 0.00 (-22.11, 16.10) | 7 (-36, 40) | 3.55 (-17.19, 19.32) | 9 (-44, 48) | 4.50 (-21.02, 22.75) |
| **Age** |  |  |  |  |  |  |
| 40-50 | 9 (-28, 34) | 7.41 (-22.47, 27.31) | 14 (-20, 37) | 11.55 (-16.13, 29.87) | 19 (-19, 45) | 15.78 (-15.61, 36.47) |
| 50-60 | 0 (-46, 35) | 0.00 (-18.77, 14.21) | 4 (-41, 38) | 1.63 (-16.64, 15.51) | 3 (-46, 40) | 1.24 (-18.52, 16.19) |
| ≥60 | 51 (0, 92) | 14.07 (0.00, 25.29) | 59 (10, 97) | 16.34 (2.73, 26.90) | 66 (6, 110) | 18.16 (1.57, 30.48) |
| **Smoking status** |  |  |  |  |  |  |
| Never smoker | 1 (-47, 38) | 0.55 (-18.41, 14.72) | 11 (-37, 46) | 4.32 (-14.21, 17.99) | 15 (-36, 57) | 5.78 (-14.13, 22.27) |
| Ever smoker | 58 (-3, 106) | 12.24 (-0.59, 22.29) | 62 (9, 108) | 13.07 (1.82, 22.70) | 70 (0, 124) | 14.68 (0.00, 26.26) |
| **Biomass fuel exposure** |  |  |  |  |  |  |
| No | -18 (-73, 29) | -4.18 (-16.71, 6.71) | -7 (-63, 36) | -1.63 (-14.61, 8.31) | -6 (-81, 48) | -1.42 (-18.59, 10.98) |
| Yes | 92 (41, 130) | 31.00 (13.77, 43.79) | 96 (49, 131) | 32.19 (16.49, 44.19) | 94 (42, 131) | 31.69 (14.18, 44.15) |
| **Occupational exposure** |  |  |  |  |  |  |
| No | -8 (-64, 34) | -2.75 (-20.97, 11.31) | 0 (-54, 41) | 0.00 (-17.86, 13.51) | -4 (-71, 44) | -1.47 (-23.55, 14.61) |
| Yes | 56 (0, 101) | 13.18 (0.00, 23.50) | 65 (12, 106) | 15.15 (2.86, 24.88) | 74 (13, 126) | 17.41 (3.15, 29.45) |
| **Ventilation** |  |  |  |  |  |  |
| No | 18 (-15, 41) | 11.39 (-9.61, 26.38) | 22 (-8, 44) | 14.50 (-5.18, 28.14) | 23 (-16, 48) | 14.64 (-10.56, 31.20) |
| Yes | 34 (-34, 91) | 5.89 (-5.83, 15.88) | 45 (-17, 102) | 7.78 (-3.00, 17.73) | 58 (-29, 117) | 10.12 (-5.08, 20.38) |
| **BMI category** |  |  |  |  |  |  |
| Normal weight or underweight | 48 (-8, 94) | 11.37 (-1.79, 22.36) | 57 (4, 100) | 13.57 (0.91, 23.81) | 61 (0, 112) | 14.48 (0.00, 26.73) |
| Overweight or obese | 2 (-53, 44) | 0.57 (-16.93, 14.03) | 8 (-45, 50) | 2.70 (-14.48, 16.09) | 14 (-51, 59) | 4.48 (-16.47, 18.82) |
| **Region** |  |  |  |  |  |  |
| Urban | -11 (-56, 23) | -4.26 (-20.95, 8.58) | -4 (-47, 30) | -1.66 (-17.53, 11.32) | 4 (-49, 43) | 1.51 (-18.41, 16.26) |
| Rural | 82 (16, 132) | 17.63 (3.50, 28.39) | 85 (26, 132) | 18.26 (5.51, 28.50) | 76 (13, 132) | 16.42 (2.76, 28.51) |

PAF: population attributable fraction; PM_1_: particulate matter ≤1.0µm; PM_2.5_: particulate matter ≤2.5µm; PM_10_: particulate matter ≤ 10µm.

**Table S8. The population-attributable fraction of asthma attributable to PMs by different strata.**

| **Strata** | **PM_1_** | | **PM_2.5_** | | **PM_10_** | |
| --- | --- | --- | --- | --- | --- | --- |
|  | **Attributable cases** | **PAF (%)** | **Attributable cases** | **PAF (%)** | **Attributable cases** | **PAF (%)** |
| **Total** | 49 (6, 84) | 15.62 (1.81, 26.72) | 54 (12, 87) | 17.18 (3.79, 27.86) | 70 (20, 108) | 22.35 (6.33, 34.35) |
| **Sex** |  |  |  |  |  |  |
| Male | 39 (8, 62) | 22.89 (4.76, 36.10) | 38 (7, 61) | 22.05 (3.84, 35.29) | 49 (14, 74) | 28.41 (7.90, 43.01) |
| Female | 11 (-23, 35) | 7.85 (-16.66, 25.14) | 17 (-16, 40) | 12.34 (-11.11, 28.58) | 22 (-18, 48) | 15.33 (-12.97, 34.29) |
| **Age** |  |  |  |  |  |  |
| 40-50 | -2 (-30, 14) | -3.07 (-58.23, 27.36) | 1 (-24, 16) | 2.76 (-46.62, 31.23) | 7 (-21, 22) | 12.85 (-40.79, 42.53) |
| 50-60 | 32 (11, 46) | 30.04 (10.17, 43.66) | 33 (13, 47) | 31.31 (12.19, 44.55) | 35 (13, 50) | 33.34 (12.00, 47.30) |
| ≥60 | 21 (-14, 45) | 13.23 (-9.14, 28.99) | 20 (-14, 45) | 13.09 (-8.66, 28.77) | 26 (-14, 55) | 16.86 (-9.15, 35.00) |
| **Smoking status** |  |  |  |  |  |  |
| Never smoker | 14 (-20, 38) | 9.18 (-13.81, 25.82) | 18 (-14, 42) | 12.26 (-9.77, 28.48) | 22 (-16, 49) | 15.18 (-10.77, 33.37) |
| Ever smoker | 37 (6, 59) | 22.38 (3.65, 35.99) | 37 (6, 58) | 22.17 (3.87, 35.42) | 49 (13, 72) | 29.51 (7.99, 43.79) |
| **Biomass fuel exposure** |  |  |  |  |  |  |
| No | 20 (-10, 43) | 11.52 (-5.62, 24.30) | 21 (-10, 44) | 11.65 (-5.96, 25.06) | 32 (-6, 58) | 18.36 (-3.34, 32.90) |
| Yes | 28 (-13, 55) | 20.75 (-9.16, 40.19) | 34 (-3, 58) | 24.88 (-2.23, 42.31) | 33 (-7, 59) | 24.35 (-5.04, 43.25) |
| **Occupational exposure** |  |  |  |  |  |  |
| No | 25 (-4, 45) | 18.26 (-3.17, 33.46) | 28 (0, 48) | 20.68 (0.00, 35.30) | 32 (-2, 56) | 23.96 (-1.68, 41.32) |
| Yes | 25 (-11, 51) | 14.00 (-6.25, 28.89) | 27 (-8, 53) | 14.91 (-4.27, 29.59) | 38 (-3, 65) | 21.34 (-1.72, 36.45) |
| **Ventilation** |  |  |  |  |  |  |
| No | 13 (-9, 26) | 18.69 (-13.38, 38.36) | 15 (-6, 28) | 21.62 (-8.07, 40.03) | 19 (-6, 33) | 26.82 (-8.38, 47.89) |
| Yes | 35 (-5, 66) | 14.33 (-1.95, 27.06) | 38 (-3, 68) | 15.56 (-1.03, 27.90) | 47 (0, 83) | 19.45 (0.00, 34.08) |
| **BMI category** |  |  |  |  |  |  |
| Normal weight or underweight | 4 (-37, 33) | 2.76 (-24.12, 21.47) | 9 (-28, 37) | 5.96 (-18.19, 23.72) | 15 (-29, 47) | 10.00 (-18.77, 30.60) |
| Overweight or obese | 39 (10, 60) | 24.63 (6.59, 37.62) | 40 (12, 61) | 25.14 (7.71, 38.18) | 49 (15, 72) | 30.95 (9.65, 45.08) |
| **Region** |  |  |  |  |  |  |
| Urban | 3 (-25, 24) | 2.62 (-20.35, 19.04) | 5 (-23, 24) | 3.95 (-18.13, 19.14) | 15 (-19, 38) | 11.80 (-15.55, 30.64) |
| Rural | 54 (17, 80) | 28.78 (9.16, 42.75) | 55 (21, 80) | 29.11 (10.98, 42.61) | 56 (18, 83) | 29.72 (9.61, 44.02) |

PAF: population attributable fraction; PM_1_: particulate matter ≤1.0µm; PM_2.5_: particulate matter ≤2.5µm; PM_10_: particulate matter ≤ 10µm.

**Table S9. Sensitivity analysis on the associations between PMs and CRDs after excluding participants with** **cardiovascular and other respiratory diseases.**

| **Diseases** | **No. of Participants** | **PM_1_** | | **PM_2.5_** | | **PM_10_** | |
| --- | --- | --- | --- | --- | --- | --- | --- |
|  |  | **OR**  **(95% CI)** | ***P* for difference test** | **OR**  **(95% CI)** | ***P* for difference test** | **OR**  **(95% CI)** | ***P* for difference test** |
| **CRD** | 7371 | 1.44 (1.25, 1.66) | - | 1.26 (1.16, 1.38) | - | 1.17 (1.10, 1.24) | - |
|  | 5345 | 1.54 (1.30, 1.83) | 0.54 | 1.31 (1.18, 1.46) | 0.53 | 1.21 (1.13, 1.31) | 0.55 |
| **COPD** | 7371 | 1.85 (1.53, 2.24) | - | 1.45 (1.29, 1.63) | - | 1.28 (1.17, 1.39) | - |
|  | 5345 | 1.86 (1.48, 2.33) | 0.95 | 1.45 (1.26, 1.67) | 1.00 | 1.27 (1.15, 1.41) | 1.00 |
| **Chronic Bronchitis** | 7371 | 1.14 (0.95, 1.37) | - | 1.12 (1.00, 1.25) | - | 1.08 (0.99, 1.17) | - |
|  | 5345 | 1.24 (0.98, 1.56) | 0.59 | 1.18 (1.02, 1.37) | 0.52 | 1.14 (1.03, 1.27) | 0.27 |
| **Asthma** | 7371 | 1.33 (1.03, 1.72) | - | 1.22 (1.04, 1.43) | - | 1.17 (1.04, 1.31) | - |
|  | 5345 | 1.31 (0.94, 1.83) | 0.93 | 1.20 (0.98, 1.48) | 0.88 | 1.19 (1.02, 1.38) | 0.92 |

The model was adjusted by age, educational level, marital status, BMI category, region, passive smoking, occupational exposure, household ventilation, biomass fuel exposure, smoking status and sex.

PM_1_: particulate matter ≤1.0µm; PM_2.5_: particulate matter ≤2.5µm; PM_10_: particulate matter ≤ 10µm.

**^*^**: the p value for the difference between the groups
